# Dose-dependent structural and dielectric response of γ-irradiated PVA/GO/FeGaInS_4_ nanocomposite films

**DOI:** 10.1039/d6ra09643c

**Published:** 2026-11-09

**Authors:** Zeynab Addayeva, Goncha Eyvazova, Namiq Niftiyev, Faik Mammadov, Oktay K. Gasymov, Rasim Aslanov, Nahida Musayeva, Hokman Mahmudov, Mustafa Muradov

**Affiliations:** a Baku State University, Nano Research Laboratory Baku Azerbaijan zeynabramazan5399@gmail.com; b Baku State University, Faculty of Physics, Department of Physics of Semiconductor Azerbaijan; c Azerbaijan State Pedagogical University Baku Azerbaijan; d Institute of Catalysis and Inorganic Chemistry Baku Azerbaijan; e Azerbaijan University of Architecture and Construction Baku Azerbaijan; f Institute of Molecular Biology, Azerbaijan Ministry of Science and Education Baku Azerbaijan; g Crystallography and Crystallophysics Laboratory, Institute of Physics, Azerbaijan Ministry of Science and Education Baku Azerbaijan; h Radiation Technologies Laboratory No. 21, Institute of Molecular Biology, Ministry of Science and Education of the Republic of Azerbaijan Baku Azerbaijan

## Abstract

Poly(vinyl alcohol) (PVA) films containing two dissimilar fillers, graphene oxide (GO) nanosheets and layered FeGaInS_4_ semiconductor crystallites, were prepared by solution casting and irradiated with ^60^Co γ-rays at 250, 500 and 1500 kGy. Since the dielectric response of dual-filler composites is governed largely by the polymer-filler interphases, the study aimed to establish how γ-irradiation modifies their polarization and charge transport and which radiation-induced processes are responsible. The films were characterized by X-ray diffraction (XRD), Fourier-transform infrared (FTIR) spectroscopy, electron paramagnetic resonance (EPR) and dielectric spectroscopy. Williamson–Hall analysis indicated that the FeGaInS_4_ crystallite size remained unchanged within experimental uncertainty at 250 kGy and decreased to about 47 nm at 500 and 1500 kGy, while broadening of the PVA (101) reflection at 1500 kGy revealed a loss of crystalline order in the matrix. EPR revealed radiation-induced radicals, whose concentration was highest at 250 kGy, together with a broad Fe-related resonance that was not affected by irradiation; during 90 days of storage, the radical signal decayed fastest at 250 kGy and most slowly at 1500 kGy, indicating the presence of comparatively stable trapped centers at the highest dose. FTIR spectra indicated dose-dependent modification of intermolecular interactions and chemical bonding in the polymer matrix. The permittivity, dielectric loss, displacement current and ac conductivity decreased at 250 and 500 kGy and increased at 1500 kGy. The frequency exponent *s* rose from 0.75 to 0.84 and fell to 0.66 at 1500 kGy, while the barrier height *W*_M_ changed from 1.10 to 1.47 eV and then to 0.94 eV, consistent with correlated barrier hopping in a crosslinked network and an increasing contribution of defect-assisted transport at the highest dose. In contrast to the GO-free FeGaInS_4_/PVA system, in which irradiation at 500 kGy enhanced the permittivity and conductivity, the GO-containing composite showed the opposite trend, suggesting that the GO-containing interphases promote crosslinking at moderate doses. Moderate γ-doses therefore favor a crosslinked network, whereas high doses lead to degradation and defect-mediated conduction.

## Abstract 2

PVA films containing two dissimilar fillers, graphene oxide (GO) nanosheets and layered FeGaInS_4_ crystallites, were prepared by solution casting and irradiated with ^60^Co γ-rays at 250, 500 and 1500 kGy.

## Graphical abstract

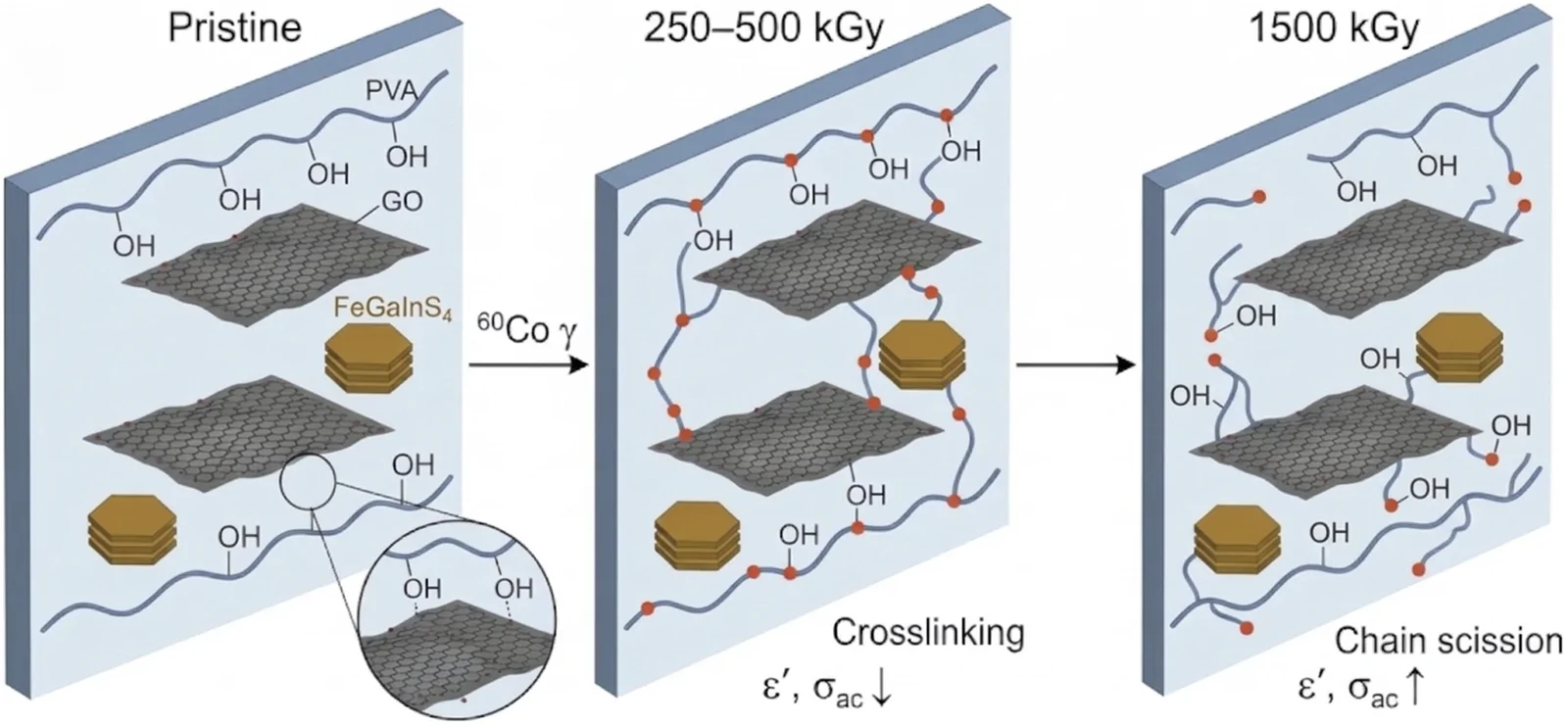

## 1 Introduction

Polymer-based nanocomposites have attracted significant attention as advanced functional dielectric materials for applications in electronics, energy storage systems, sensors, insulating components,^1^ and radiation-resistant devices.^2^ Their low density, mechanical flexibility, easy processability, and tunable electrical properties make them highly promising for modern technological applications.^3^ The dielectric and electrical behavior of polymer systems is strongly influenced by their internal structure,^4^ including crystallinity, molecular ordering, interfacial morphology, and the distribution of filler particles.^5^ Therefore, controlled structural modification of polymer materials is considered one of the most effective strategies for tailoring their functional properties.^6^

The incorporation of nanoscale fillers into polymer matrices has become an efficient approach for improving dielectric performance.^7^ Nanofillers can introduce additional polarization centers,^8^ modify charge transport pathways,^9^ and enhance interfacial interactions within the composite structure.^10^ In particular, two-dimensional graphene oxide (GO) has emerged as an attractive nanofiller because of its large specific surface area, layered structure, and abundance of oxygen-containing functional groups.^10^ These characteristics promote strong interactions with polar polymer matrices and significantly influence dielectric polarization and electrical conductivity. Moreover, the coexistence of conductive sp^2^ domains and insulating sp^3^ regions in GO^11^ favors interfacial Maxwell–Wagner–Sillars polarization, especially at low frequencies.^12^

Besides compositional modification, ionizing radiation is widely used as an effective method for controlling the structure and physical properties of polymer materials.^13^ Gamma irradiation can induce several competing processes in polymer nanocomposites, including chain scission, crosslinking, oxidation, defect formation, and interfacial rearrangement.^14^ The predominance of these mechanisms depends on the absorbed radiation dose, the chemical nature of the polymer matrix, the filler type,^15^ and the irradiation environment. As a consequence, gamma irradiation may either enhance or deteriorate dielectric behavior through changes in crystallinity,^16^ molecular mobility, defect concentration, and charge carrier dynamics.^17^

The radiation response of polymer dielectrics is of direct practical relevance. Such materials serve as cable insulation in nuclear power plants, where the dose accumulated over the service life can reach the MGy range,^18^ as insulating components in spacecraft electronics,^19^ and in medical devices that undergo radiation sterilization, typically at doses of 25 kGy or higher.^20^ At the same time, γ-irradiation induces crosslinking without chemical initiators, a route widely used, for example, in hydrogel formation,^21^ so irradiation can also be employed deliberately to tailor the dielectric properties of polymer composites.

PVA was selected as the polymer matrix in the present work due to its excellent film-forming ability, semi-crystalline structure, and high concentration of polar hydroxyl groups. The presence of –OH groups makes PVA highly sensitive to intermolecular interactions and structural modifications induced by fillers or irradiation.^22^ Previous studies have shown that gamma irradiation can significantly alter the crystalline structure and dielectric response of PVA-based systems through radiation-induced crosslinking^23^ and chain fragmentation processes.^24^ Furthermore, the incorporation of GO into the PVA matrix can strengthen^25^ interfacial polarization effects^26^ and modify the dielectric relaxation behavior of the composite.^27,28^ For example, in PVA/Se nanocomposites irradiated at 20–100 kGy, the permittivity and ac conductivity were found to increase monotonically with dose, owing to radiation-induced defects and localized states, and carbonyl groups were formed at higher doses.^29^ The dielectric response at considerably higher doses, where extensive crosslinking and subsequent chain scission of the matrix are expected, has received much less attention.

Multinary metal chalcogenides, including quaternary compounds such as Cu_2_ZnSnS_4_, have attracted growing interest because their electrical and optical properties can be tuned through composition and structure, and they can exhibit pronounced frequency-dependent and nonlinear responses.^30^ Among them, layered chalcogenides are of particular interest owing to their anisotropic crystal structure and tunable electrical, optical and dielectric properties.^31–33^ Among them, FeGaInS_4_ is considered a promising layered semiconductor material with reported band-gap values around 1.4–1.5 eV and temperature-dependent electrical conductivity.^34,35^ The increase in conductivity with temperature is commonly associated with thermally activated hopping transport between localized states.^36^ In addition, the presence of Fe ions may contribute to defect-related and magnetic effects,^37^ making FeGaInS_4_ an attractive multifunctional filler for polymer nanocomposites.^38,39^

Despite the increasing interest in radiation-modified polymer nanocomposites, the combined influence of GO, FeGaInS_4_ layered crystals and γ-irradiation on the dielectric behavior of PVA-based systems has not been sufficiently investigated. The PVA/GO/FeGaInS_4_ system has so far been studied only in the non-irradiated state,^40^ whereas the γ-irradiation response has been reported only for the binary FeGaInS_4_/PVA composite (500–2500 kGy).^41^ In the binary composite, irradiation at 500 kGy was found to increase the permittivity and conductivity, which was attributed to radiation-induced crosslinking.^41^ In a dual-filler system, however, the dielectric response is controlled to a large extent by the interphases formed between the polymer and the two chemically and electronically dissimilar fillers. Because these interphases contain oxygen-functionalized GO surfaces and semiconductor-polymer boundaries, their response to ionizing radiation is expected to differ from that of the bulk matrix, and the behavior of the irradiated ternary composite cannot be predicted from that of its binary counterparts. To our knowledge, the effect of γ-irradiation on such dual-filler PVA composites has not been reported.

In this work, PVA/GO/FeGaInS_4_ films were prepared by solution casting and irradiated with ^60^Co γ-rays at 250, 500 and 1500 kGy, with the aim of establishing how irradiation modifies their polarization and charge transport and which radiation-induced processes are responsible. The doses were chosen to extend the range examined in our previous study towards lower doses, with a closer spacing in the 250–500 kGy range, where radical formation, recombination and crosslinking are expected to compete most strongly; two doses (500 and 1500 kGy) were kept identical to those of the earlier study to permit a direct comparison.^41^ Because the dielectric changes induced by irradiation originate from radical-mediated reactions, EPR spectroscopy was used to detect the radiation-induced paramagnetic centres directly and to follow their stability over time. Structural and chemical changes were examined by XRD and FTIR, and the dielectric response was analysed in terms of permittivity, loss, displacement current and ac conductivity, with particular attention to interfacial polarization.

## 2 Materials and methods

### 2.1 Synthesis of FeGaInS_4_/PVA/GO composites

To prepare the FeGaInS_4_/PVA/GO composites, FeGaInS_4_ crystals and graphene oxide (GO) were first synthesized independently, then incorporated into a 5 wt% polyvinyl alcohol (PVA) solution using a casting solution technique.

GO was produced using a modified version of Hummer's method. In this process, 3 g of graphite powder and 1.5 g of sodium nitrate were mixed in a 500 mL beaker. While maintaining the mixture in an ice bath, 70 mL of concentrated sulfuric acid (H_2_SO_4_) was slowly added, and the resulting solution was stirred for 1 hour. Potassium permanganate (KMnO_4_) was then introduced gradually, keeping the reaction temperature below 20 °C. After stirring for 3 hours under controlled conditions, the mixture was further stirred at 35 °C for an additional hour. Next, 150 mL of water was added dropwise, causing the viscosity to increase and the temperature to rise to 98 °C, where it was held for 30 minutes. Following this, the mixture was diluted with 300 mL of water, stirred for 1 hour, and treated with 15 mL of 30 wt% hydrogen peroxide (H_2_O_2_) for 30 minutes. The resultant material was filtered and washed with a 1 : 10 hydrochloric acid (HCl) to distilled water solution (250 mL) to eliminate remaining metal ions. The filtered product was air-dried at room temperature, redispersed in distilled water, sonicated, centrifuged, filtered again, and finally dried under ambient conditions.^42–45^

FeGaInS_4_ crystals were synthesized by sealing accurately weighed precursor materials in a vacuum quartz ampoule. This ampoule was placed in a two-zone furnace, where it underwent a thermal process at 800 K for 100 hours to promote crystallinity.^31–33^

For the polymer matrix, a 3 wt% aqueous PVA solution was prepared. GO (0.8 wt% relative to PVA) was added to this solution, and the dispersion was ultrasonicated for 20 min. FeGaInS_4_ crystal powder (3 wt% relative to PVA) was then added under mechanical stirring, and the mixture was ultrasonicated for a further 15 min to obtain a homogeneous dispersion. The mixture was cast into a Petri dish and dried at 25 °C for 48 h. The resulting flexible film, with a thickness of 165 µm, was peeled off and cut into four pieces: one was kept as the non-irradiated reference, and the other three were irradiated at 250, 500 and 1500 kGy. Since all samples originate from the same film, the differences between them can be attributed to irradiation rather than to batch-to-batch variations in composition or filler dispersion. The preparation procedure is shown schematically in Fig. 1. For irradiation, the film pieces were placed in borosilicate glass tubes, which were flame-sealed in air. γ-Irradiation was carried out with a ^60^Co source (γ-quanta of 1.17 and 1.33 MeV; mean energy ≈1.25 MeV) at a dose rate of 4.23 kGy h^−1^ and room temperature, up to absorbed doses of 250, 500 and 1500 kGy.

**Fig. 1:**
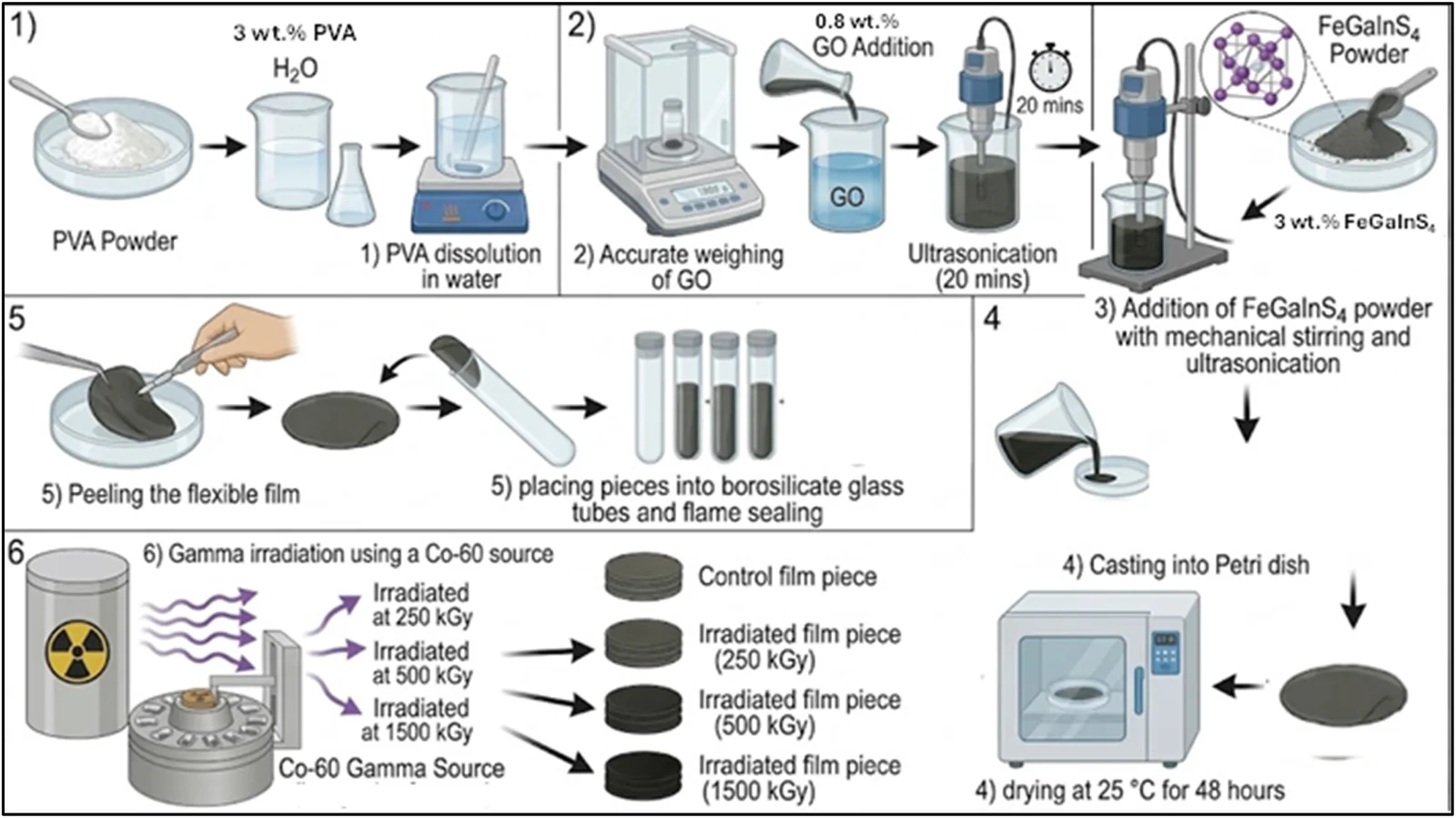
Schematic of the preparation of FeGaInS_4_/PVA/GO films and their γ-irradiation. A single film was cut into four pieces (N0–N3); N0 was kept as the non-irradiated reference. Image generated by Google Gemini.

### 2.2 Characterization of FeGaInS_4_/PVA/GO composites

The samples analysis with EPR, XRD, FTIR and dielectric spectroscopy. X-ray Diffraction (XRD): the phase purity and crystalline structure were analyzed using a Bruker D8 phaser powder X-ray diffractometer with Cu Kα radiation. Data were collected in the 2*θ* range of 5–80° with a step size of 0.02°.

FTIR spectra were recorded in transmission mode using KBr pellets on a Shimadzu IR Affinity-1 spectrometer in the range 400–4000 cm^−1^.

EPR spectra were recorded using a Bruker ELEXSYS E580 spectrometer operating at X-band frequency and equipped with a room-temperature measurement accessory.

The dielectric properties were measured with an E7-20 RLC meter (basic accuracy ±0.1% for capacitance and ±0.001 for the loss factor) in the frequency range of 120 Hz^−1^ MHz and the temperature range of 20–100 °C, using electrodes 5 mm in diameter. The film thickness was measured with a dial thickness gauge (scale division 0.01 mm) at 5 points and averaged, giving *d* = 165 ± 5 µm. The overall uncertainty of the derived quantities (*ε*′, *σ*_ac_ and *J*_d_), dominated by the uncertainties of the film thickness and electrode area, is estimated to be ±4%.

### 2.3 Methods

#### 2.3.1 Electron paramagnetic resonance (EPR) analysis

Electron paramagnetic resonance (EPR) measurements were performed at room temperature using a Bruker ELEXSYS E580 spectrometer operating in the X-band region. The EPR spectra were recorded for the pristine and γ-irradiated PVA/GO/FeGaInS_4_ nanocomposites immediately after irradiation and after approximately 90 days of storage.

The resonance magnetic field (*B*_r_) was determined from the center position of the EPR signal according to:1
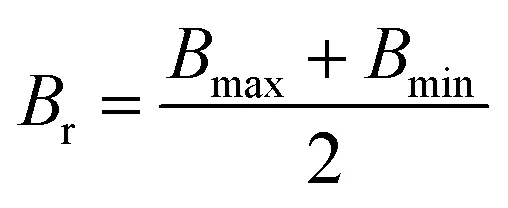
where (*B*_max_) and (*B*_min_) are the magnetic field positions corresponding to the maximum and minimum amplitudes of the first-derivative EPR signal.^46^

The spectroscopic splitting factor (*g*) was calculated using:2
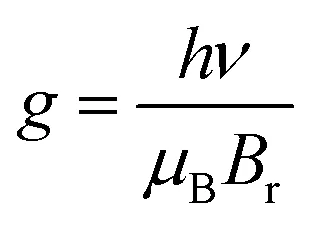
where (*h*) is Planck's constant, (*ν*) is the microwave frequency, and (*µ*_B_) is the Bohr magneton.

The peak-to-peak linewidth (Δ*B*_pp_) was calculated as:

3 Δ*B*_pp_ = |*B*_max_ − *B*_min_|

To evaluate the radiation-induced free radicals, the resonance signal located in the 3300–3420 G magnetic field region was analyzed separately.^47^ The peak-to-peak signal intensity was determined from:

4 *I*_pp_ = *I*_max_ − *I*_min_

where (*I*_max_) and (*I*_min_) represent the maximum and minimum amplitudes of the derivative EPR signal, respectively.^48^

For comparative analysis, the relative radical concentration was estimated using the relation:

5 *A*_rel_ ∼ *I*_pp_ (Δ*B*_pp_)^2^

where (*A*_rel_) is proportional to the integrated EPR signal intensity and was used as a measure of the relative concentration of radiation-induced paramagnetic centers.^49^ Since all spectra were recorded under identical experimental conditions, the calculated values were normalized with respect to the sample exhibiting the highest radical concentration.

The temporal stability of the radiation-induced radicals was evaluated by comparing the EPR spectra recorded immediately after irradiation with those recorded after approximately 90 days of storage at room temperature.

#### 2.3.2 XRD

##### 2.3.2.1 Crystallite size and micro strain

XRD spectra are widely used to determine crystallite size, structural defects, and internal stresses in materials. The crystallite size of a sample can be calculated using the Scherrer equation, which is expressed as:^50^6
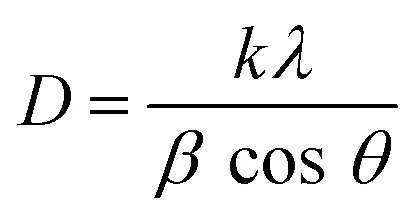
where: *D* represents the average size of ordered (crystalline) domains, which can be smaller than or equal to the grain size, and the grain size can be smaller than or equal to the particle size. *k* is a dimensionless shape factor with a standard value of about 0.9, usually close to 1, depending on the shape of the crystallites. *λ* is the wavelength of the X-rays. *β* is the line broadening at half maximum intensity (FWHM) after calculating the instrumental broadening, expressed in radians. This value is sometimes called Δ(2*θ*). *θ* is the Bragg angle in radians.

Additionally, the micro strain (*ε*), which quantifies the lattice distortions, can be estimated from the peak broadening using the following relation:^51^7
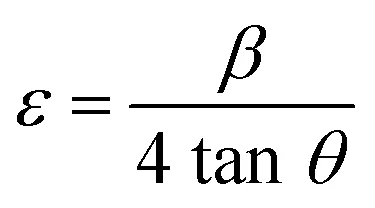
where: *β* is the FWHM of the peak in radians. *θ* is the Bragg angle in radians.

Because the Scherrer equation attributes the entire line broadening to the finite crystallite size, the Williamson–Hall (W–H) method was used, which separates the size- and strain-induced contributions to the broadening.^50,51^

The W–H method distinguishes the contributions of size-induced and strain-induced broadening through a linear plot of *β* cos *θ versus* 4 sin *θ*. The slope of this plot yields the micro strain (*ε*), while the intercept provides the crystallite size (*D*). This dual analysis makes the W–H method especially suitable for materials where strain effects cannot be ignored, such as nanostructured materials or those with significant lattice defects. Reflections whose fitted FWHM coincided with the step size (0.02°) were excluded from the analysis, and the uncertainties were obtained from the standard errors of the linear fits.

#### 2.3.3 Dielectric spectroscopy

In polymer materials, two primary charge transport models are observed: quantum mechanical tunnelling (QMT) and correlated barrier hopping (CBH).^52^

According to the CBH (correlated barrier hopping) model, the electrons in the electric field:8
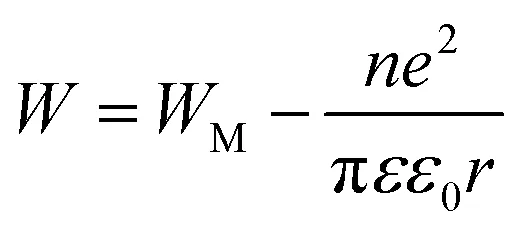
Here, *W*_M_ is the maximum height of the potential barrier, *ε* is the dielectric constant of the material, *ε*_0_ is the vacuum permittivity, *r* is the distance between the two states of the charge carriers, and *n* is the number of electrons undergoing the hopping process (which can take values of 1 for polaron and 2 for bipolar processes).^52^

For the electrical conductivity in the CBH model, the formula is:9
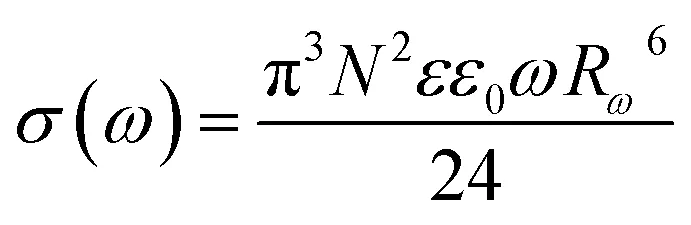
where *N* is the density of pairs of states through which the charge carriers hop.

The relationship between the hopping distance *R*_ω_ and the potential barrier *W*_M_ is given by:10
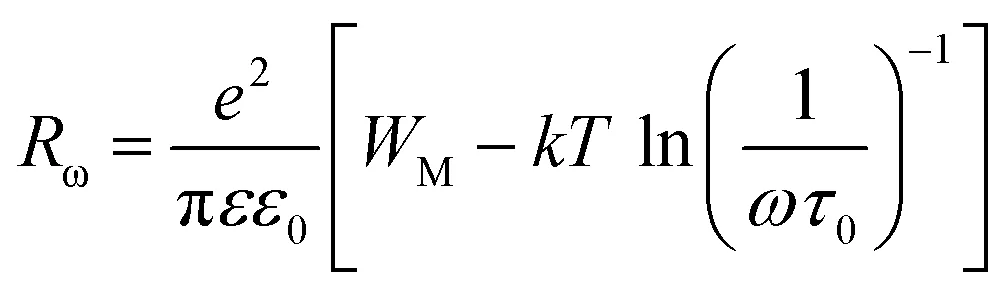
where *τ*_0_ – is the characteristic relaxation time, which is the inverse of the phonon frequency *ν*_f_.

The expression for the exponent *s* as a function of the potential barrier height *W*_M_:11
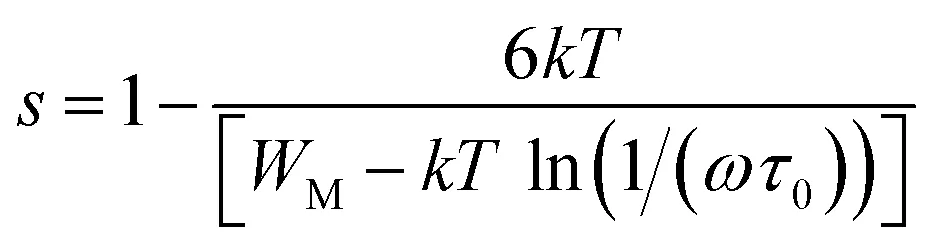


Eqn (11) can be simplified to:12
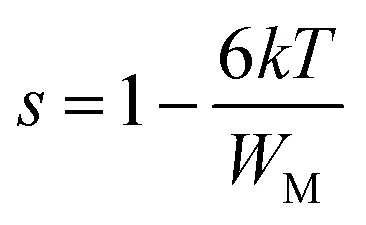


The theoretical study of the temperature dependence of conductivity based on Debye analysis of frequency dependence was considered in, where the temperature dependence of conductivity was determined as:^52–54^13
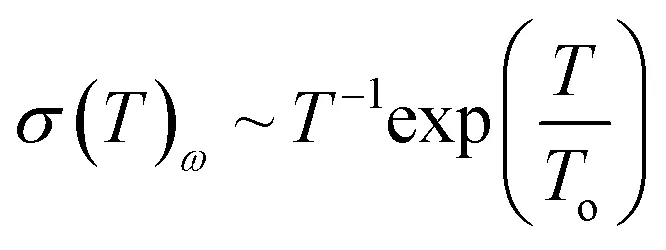
where *T*_o_ is the characteristic temperature. According to eqn (13), for temperatures *T* > *T*_o_ the dependence of electrical conductivity on temperature should yield a straight line in the ln(*σ*·*T*) ∼ *f*(*T*) coordinates.^52^

In the studied temperature range, electrical conductivity follows an activation mechanism and is described by the following equation:14
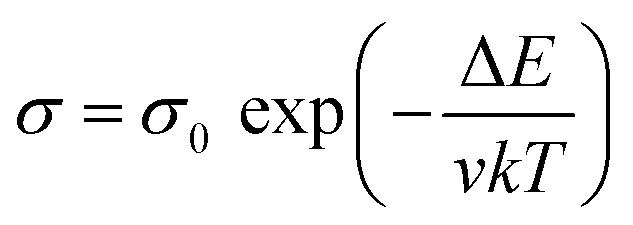
where Δ*E* is the activation energy, and *k* is the Boltzmann constant.

##### 2.3.3.1 Displacement current analysis

To evaluate the contribution of polarization processes to the AC electrical response, the displacement current density (*J*_d_) was calculated using Maxwell's displacement current formalism.^55–57^ Under an alternating electric field, the total current density consists of conduction and displacement current components and can be expressed as:

15 *J* = *J*_c_ + *J*_d_

where (*J*_c_) is the conduction current density and (*J*_d_) is the displacement current density.

The displacement current density was determined according to:

16 *J*_d_ = *ωε*′*ε*_0_*E*

where (*ω*) is the angular frequency of the applied electric field, (*ε*_0_) is the permittivity of free space ((8.854 × 10^−12^) F m^−1^), (*ε*′) is the real part of the dielectric permittivity, and (*E*) is the electric field strength.^55^ The electric field was calculated from:17
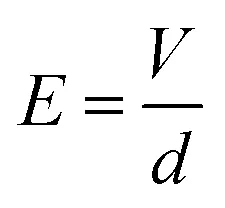
where (*V*) is the applied AC voltage (1 V) and (*d*) is the sample thickness.

Substituting the electric field expression into the displacement current equation gives:18
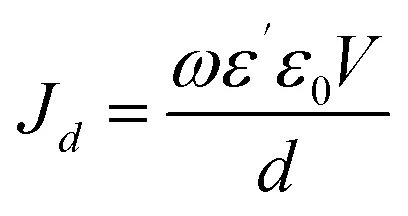


The calculated displacement current density was used to assess the contribution of polarization-related processes to the overall AC electrical response of the PVA/FeGaInS_4_/GO composites. The obtained values were compared with the frequency-dependent conductivity behavior to distinguish polarization effects from charge transport processes.

### 2.4 Nomenclature of samples

Throughout the manuscript, the composite samples are denoted as follows:

N0 – 3 wt% FeGaInS_4_/0.8 wt%GO/PVA (unirradiated).

N1 – 3 wt% FeGaInS_4_/0.8 wt%GO/PVA irradiated with 250 kGy.

N2 – 3 wt% FeGaInS_4_/0.8 wt%GO/PVA irradiated with 500 kGy.

N3 – 3 wt% FeGaInS_4_/0.8 wt%GO/PVA irradiated with 1500 kGy.

## 3 Results and discussion

### 3.1 EPR analysis

Electron paramagnetic resonance (EPR) spectroscopy was employed to investigate radiation-induced paramagnetic defects and free-radical formation in PVA/GO/FeGaInS_4_ nanocomposites. Fig. 2 presents the EPR spectra of the non-irradiated and γ-irradiated samples recorded immediately after irradiation and after approximately 90 days of storage.

**Fig. 2:**
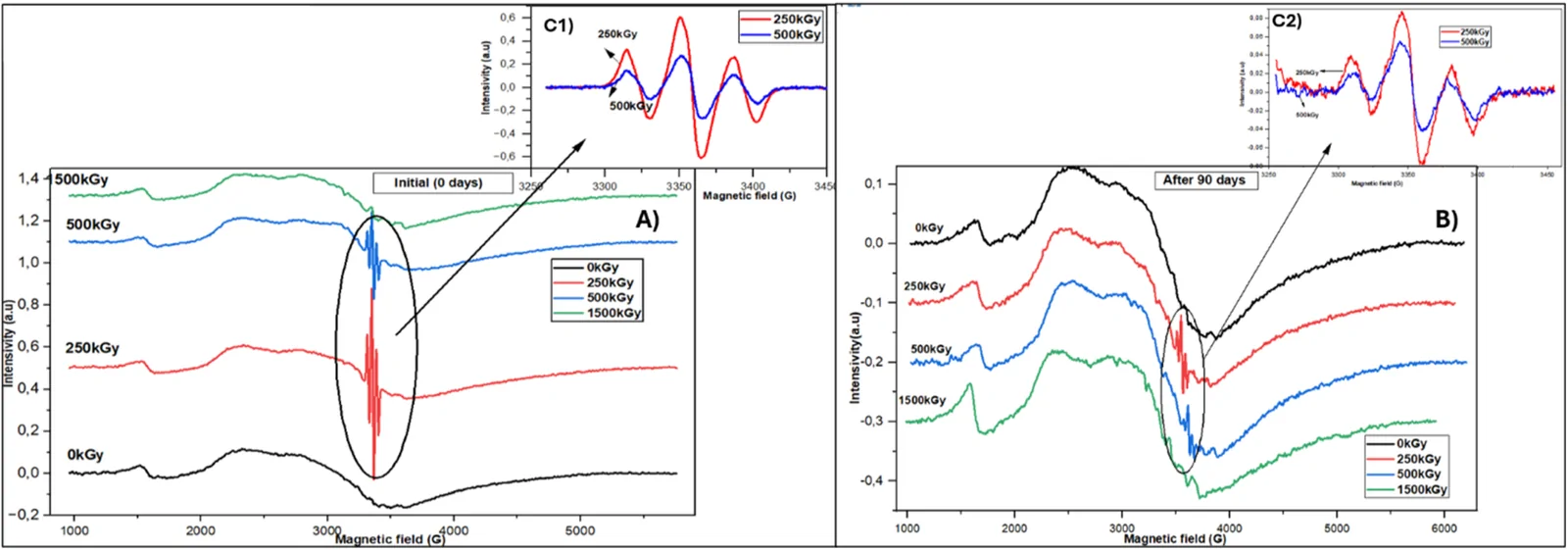
X-band EPR spectra of PVA/FeGaInS_4_/GO composites exposed to γ-radiation doses of 0, 250, 500, and 1500 kGy, recorded (A) immediately after irradiation and (B) after 90 days of storage at room temperature. Insets C1 and C2 present enlarged views of the radical-related resonance centered at *g* ≈ 2.008 (≈3340 G) for the 250 and 500 kGy samples, highlighting the evolution of radiation-induced radicals during storage.

All spectra exhibit a broad resonance signal associated with Fe-related paramagnetic centers originating from the FeGaInS_4_ phase. The position and overall shape of this resonance remain largely unchanged after irradiation, indicating that γ-irradiation does not significantly alter the local magnetic environment of these centers. This observation suggests that the FeGaInS_4_ crystalline phase remains structurally stable within the investigated irradiation dose range. The EPR parameters of the broad Fe-related resonance are summarized in Table 1.

**Table 1:** EPR parameters of the broad Fe-related resonance in PVA/GO/FeGaInS_4_ nanocomposites exposed to different γ-irradiation doses

Sample	Δ*B*_pp_ (G)
0 kGy	642
250 kGy	890
500 kGy	858
1500 kGy	1010

The peak-to-peak linewidth (Δ*B*_pp_) increases from 642 G for the pristine sample to 890, 858, and 1010 G for the 250, 500, and 1500 kGy samples, respectively. The broadening of the resonance suggests increased magnetic inhomogeneity and local structural disorder in the vicinity of Fe-related paramagnetic centers.^58–60^ However, the persistence of this broad signal after irradiation and long-term storage confirms that these centers are stable and are not directly associated with transient radiation-induced radicals.^61^ In addition, a weak resonance at *g* ≈ 4.3 (≈1550–1650 G) is observed in all spectra. This signal is characteristic of high-spin Fe^3+^ ions in sites of strong rhombic distortion.^58,62^ Since iron is nominally divalent in FeGaInS_4_, and Fe^2+^ is usually EPR-silent at X-band and room temperature, this signal indicates that a small fraction of the iron is present as Fe^3+^, most likely at the surfaces or defect sites of the FeGaInS_4_ particles and at the filler–matrix interface.

In addition to the broad Fe-related resonance, an additional narrow signal centered in the 3340–3360 G region is observed in the irradiated samples. To examine this feature in greater detail, EPR measurements were performed over a narrower magnetic field range, and the corresponding spectra are presented in Fig. 2(C1 and C2). The resonance field of this signal was found at approximately 3343–3344 G, corresponding to a *g*-factor of 2.008–2.009, which is characteristic of radiation-induced organic free radicals. The EPR parameters extracted from this resonance are listed in Table 2.

**Table 2:** EPR parameters and relative radical content of the radiation-induced radical resonance measured immediately after γ-irradiation, normalized to the 250 kGy sample at the same measurement time (100%)

Sample	*B*_max_ (G)	*B*_min_ (G)	Δ*B*_pp_ (G)	*B*_r_ (G)	*I*_pp_	Relative radical amount
0 kGy	3331.9	3364.7	32.8	3348.3	0.019	∼8%
250 kGy	3350.6	3364.7	14.1	3357.7	0.933	100%
500 kGy	3350.6	3369.4	18.8	3360.0	0.421	∼52%
1500 kGy	3350.6	3402.2	51.6	3376.4	0.049	∼18%

The appearance of this signal is attributed to free radicals generated within the polymer matrix and at the polymer-filler interfaces as a consequence of ionization and bond-cleavage processes induced by γ-irradiation. No six-line hyperfine pattern characteristic of Mn^2+^, a common residual impurity of GO prepared by the Hummers' method, was observed in the 3100–3600 G region, indicating that the Mn content is below the EPR detection limit and does not contribute to this signal.^63^ The intensity of the radical-related resonance depends strongly on the irradiation dose and reaches its maximum for the 250 kGy sample. The peak-to-peak linewidth (Δ*B*_pp_) increases from 14.1 G for the 250 kGy sample to 18.8 G for the 500 kGy sample, indicating a broader distribution of local magnetic environments and enhanced spin–spin interactions with increasing dose.

The relative radical concentration was estimated from the EPR signal intensity and linewidth. The highest radical concentration was observed for the 250 kGy sample. Compared with this sample, the relative radical content decreased to approximately 52% for the 500 kGy sample and to 18% for the 1500 kGy sample. This behavior indicates that a considerable fraction of the initially generated radicals participates in recombination reactions and is consumed during subsequent radiation-induced structural transformations. The reduction of the radical signal at high irradiation doses suggests that radical recombination and post-irradiation stabilization processes become increasingly important as the absorbed dose increases.^64^

To evaluate the stability of the irradiation-induced radicals, the EPR measurements were repeated after approximately 90 days of storage. The parameters extracted from these spectra are summarized in Table 3.

**Table 3:** EPR parameters and relative radical content of the radiation-induced radical resonance measured after 90 days of storage, normalized to the 250 kGy sample at the same measurement time (100%)

Sample	*B*_max_ (G)	*B*_min_ (G)	Δ*B*_pp_ (G)	*B*_r_ (G)	*I*_pp_	Relative radical amount
0 kGy	3345.9	3369.4	23.4	3357.7	0.0215	∼17%
250 kGy	3350.6	3364.7	14.1	3357.7	0.1261	100%
500 kGy	3350.6	3369.4	18.8	3360.0	0.0787	∼62%
1500 kGy	3345.9	3378.8	32.8	3362.3	0.0313	∼25%

After storage, the radical resonance remained detectable in all irradiated samples, but its intensity was considerably lower than that measured immediately after irradiation, confirming the metastable nature of the radiation-induced radicals. The 250 kGy sample still exhibited the highest radical content and was again used as the reference (100%), whereas the relative radical content of the 500 and 1500 kGy samples was approximately 62% and 25%, respectively.

Table 4 compares the signal intensity of each sample before and after storage. The remaining signal amounted to 13.5%, 18.7% and 64.0% of the initial intensity for the 250, 500 and 1500 kGy samples, respectively.

**Table 4:** Evolution of the radical-related EPR signal intensity during 90 days of storage. The remaining signal is given as *I*_pp_ (90 d)/*I*_pp_ (0 d) × 100% for each sample; unlike Tables 2 and 3, these values are not normalized to the 250 kGy sample

Sample	Day 0 (*I*_pp_)	After 90 days (*I*_pp_)	Remaining signal (%)
250 kGy	0.933	0.126	13.5
500 kGy	0.421	0.079	18.7
1500 kGy	0.049	0.031	64.0

It should be noted that the relative radical contents in Tables 2 and 3 were normalized independently to the 250 kGy sample within each measurement series and therefore describe the distribution of radicals among the samples at a given time, whereas Table 4 reflects the actual temporal evolution of the signal of each sample.

Although the absolute signal intensity decreased for all irradiated samples, the relative distribution of the remaining radicals changed: the relative radical content of the 500 kGy sample increased from approximately 52% to 62%, and that of the 1500 kGy sample from 18% to 25%. This increase does not indicate the formation of additional radicals. It reflects the slower decay of these samples relative to the 250 kGy reference, which retained only 13.5% of its initial signal, whereas the 1500 kGy sample retained approximately 64%.

The faster decay observed for the 250 kGy sample, which contained the highest initial radical concentration, is consistent with bimolecular recombination, a process reported to follow second-order kinetics in irradiated polymers.^65,66^ In contrast, the slow decay at 1500 kGy indicates the presence of comparatively stable, trapped paramagnetic centers. The narrowing of the resonance and the shift of its center during storage further suggest that the signal consists of at least two components, the broader of which decays more rapidly. Part of the persistent component may originate from GO. γ-Irradiation partially reduces GO and restores its sp^2^-conjugated domains,^67^ and the reduction of GO has been shown to generate defect-related singly occupied states within the π–π* gap, which give rise to paramagnetism.^68^ Because such spins are bound to structural defects, they are not expected to decay through the bimolecular recombination that governs radicals on polymer chains. A further contribution may arise from the polymer matrix itself. Conjugated unsaturated sequences can form in irradiated PVA,^64,69^ and polyenyl radicals delocalized along such sequences have been found to persist for years when trapped in rigid crystalline regions.^65^ More generally, restricted segmental mobility in densely crosslinked networks is known to hinder bimolecular radical termination.^66^ The EPR response of GO is, however, also sensitive to adsorbed water and oxygen.^70^ Since PVA is hygroscopic, changes in the moisture content of the films during storage may have contributed to the evolution of the GO-related part of the signal. An unambiguous assignment of the paramagnetic centers would require spectral simulation and power-saturation measurements, which are beyond the scope of the present study.^71–73^

In contrast to the radical resonance, the broad Fe-related resonance remained essentially unchanged after storage, confirming its association with stable paramagnetic centers of the FeGaInS_4_ phase. The marked decrease of the radical resonance with time confirms that it originates predominantly from radiation-induced radicals rather than from stable intrinsic defects of the composite.^64,74^

### 3.2 XRD analysis

To investigate the structural changes induced by gamma irradiation, the irradiated and non-irradiated nanocomposite samples were analyzed by X-ray diffraction (XRD). Fig. 3 presents the XRD patterns of the samples exposed to different gamma irradiation doses.

**Fig. 3:**
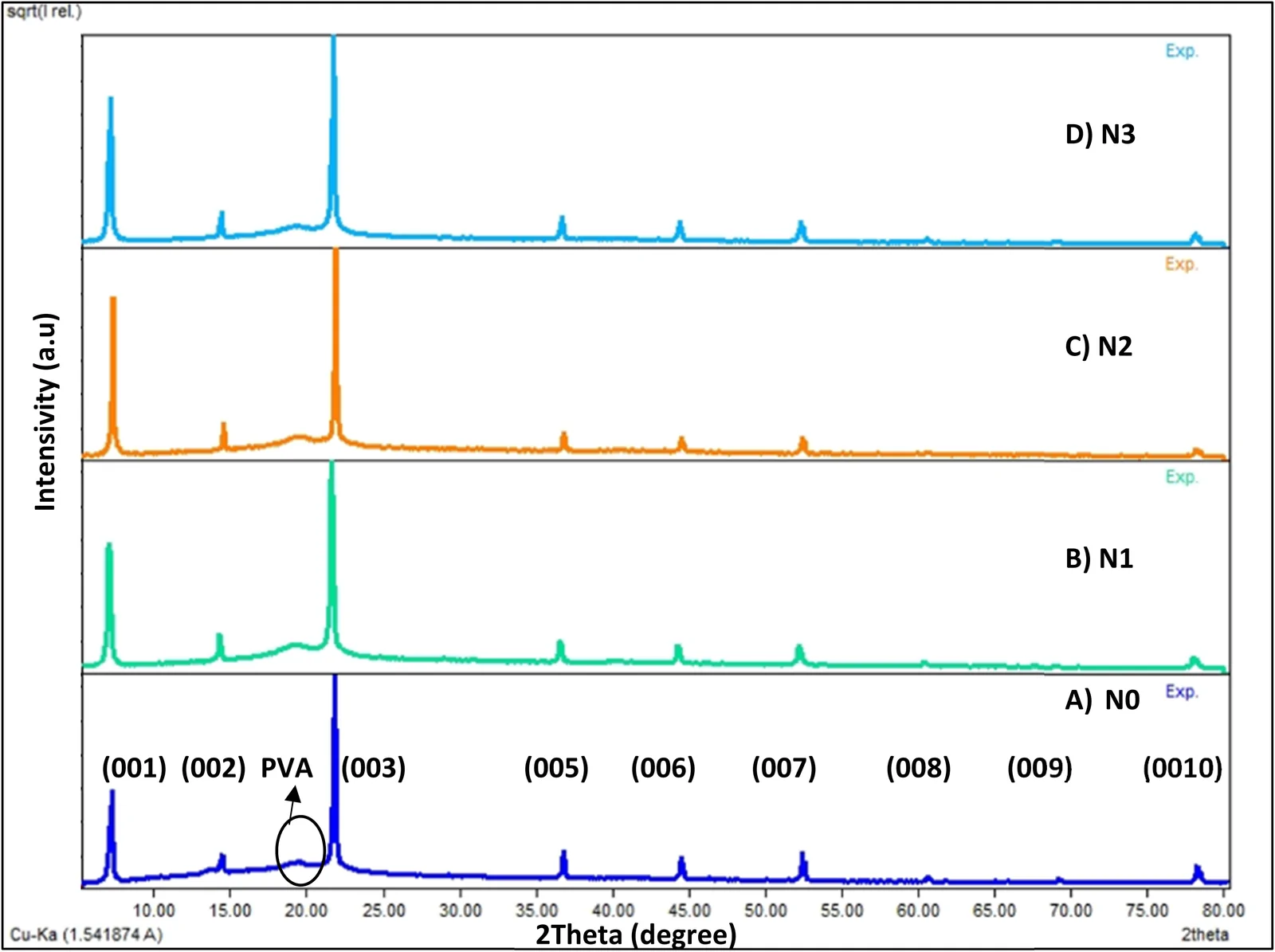
XRD patterns of (A) N0, (B) N1, (C) N2, and (D) N3 samples.

The X-ray diffraction patterns of the synthesized composites exhibit characteristic diffraction peaks located at 2*θ* = 7.01°, 14.5°, 21.7°, 36.7°, 44.2°, 52.12°, 60.4°, 69°, and 78.1°, corresponding to the (001), (002), (003), (005), (006), (007), (008), (009), and (00 10) crystallographic planes of FeGaInS_4_ (space group *P*3*m*1, No. 156), respectively, in agreement with previously reported data.^31–33^ The presence of these well-defined reflections confirms that FeGaInS4 preserves its crystalline structure within the composite matrix. Since only (00l) reflections are observed, the layered crystallites are preferentially oriented with their basal planes parallel to the film surface. Because the dielectric measurements were performed perpendicular to the film plane, the dielectric response reported here corresponds to the direction normal to the basal planes of the oriented crystallites, and the in-plane response may differ owing to the structural anisotropy of FeGaInS_4_.

In addition to the FeGaInS_4_-related reflections, all composite samples show a broad diffraction peak centered around 2*θ* ≈ 19.31°, corresponding to the semicrystalline nature of PVA and associated with the (101) diffraction plane of the polymer matrix.^34,41,75^ The persistence of this broad peak confirms the successful incorporation of PVA into the composite structure.

No characteristic diffraction peak corresponding to graphene oxide was detected in the XRD patterns. This behavior is commonly reported for GO dispersed in polymer matrices and indicates that GO exists in a highly exfoliated and disordered form inside the PVA matrix.^34,76^ Strong interfacial interactions between GO nanosheets and PVA chains suppress the characteristic GO (001) reflection near 2*θ* ≈ 10°, suggesting homogeneous dispersion of GO within the polymer matrix.

Generally, the XRD results confirm that the FeGaInS_4_ crystalline phase remains structurally stable after hybridization and irradiation, while GO is distributed in an exfoliated state within the PVA matrix, resulting in a homogeneous hybrid nanocomposite structure.

To further investigate the influence of gamma irradiation on the microstructural properties of the composites, Williamson–Hall (W–H) analysis was performed (Fig. 4).

**Fig. 4:**
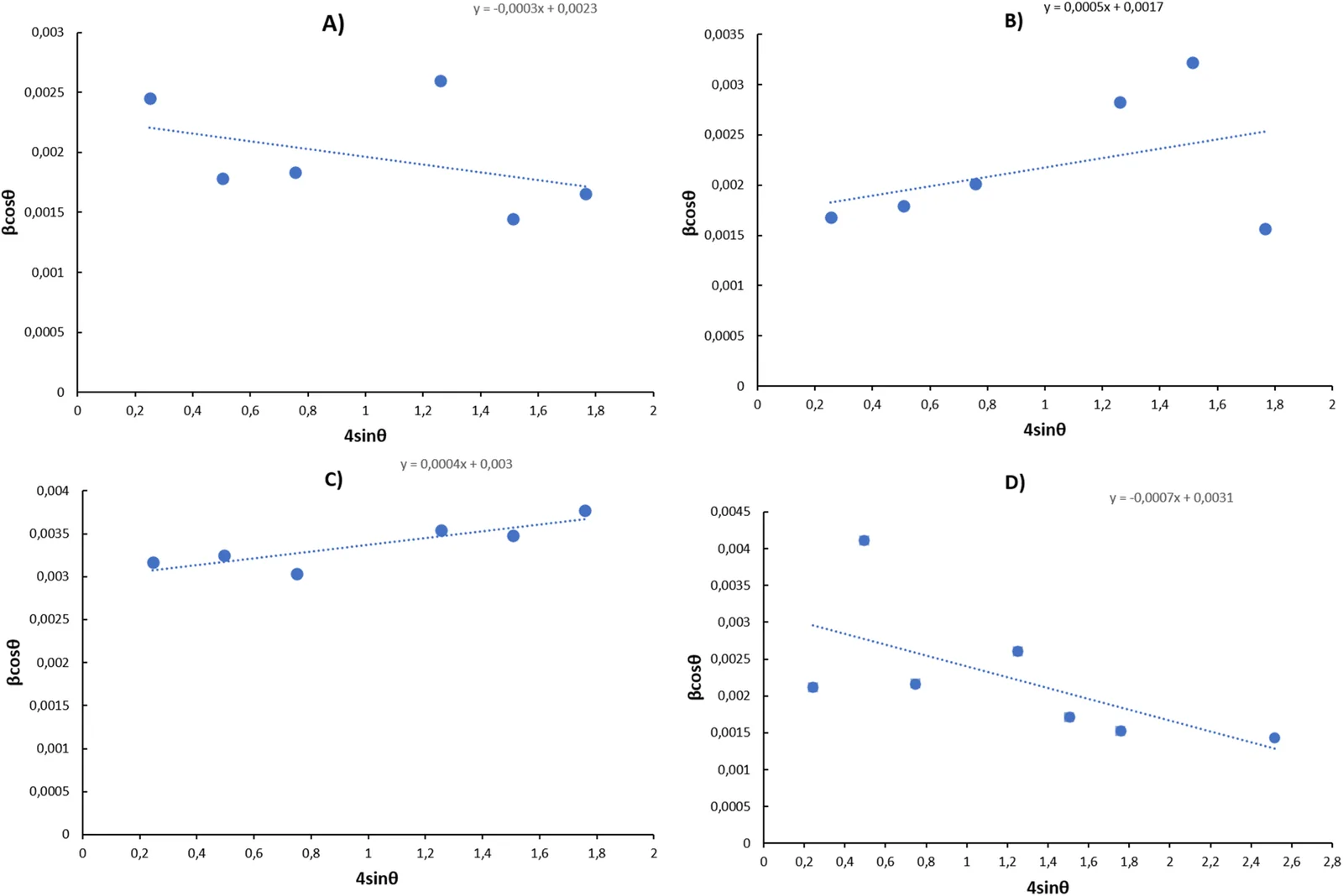
The W–H plots of (A) N0, (B) N1, (C) N2, and (D) N3 samples.

The crystallite size, microstrain, and dislocation density values calculated using the Williamson–Hall method are summarized in Table 5.

**Table 5:** Microstructural parameters of FeGaInS_4_ obtained from Williamson–Hall analysis and FWHM of the PVA (101) reflection for the irradiated composites

Sample	FWHM (PVA∼19.31°)	Crystallite size −*D*_W–H_ (nm)	Microstrain *ε*(×10^−4^)	Dislocation density *δ*(×10^−3^ nm^−2^)
0 kGy	2.08°	63 ± 11	−3.3 ± 3.5	0.25 ± 0.09
250 kGy	2.40°	85 ± 30	4.7 ± 5.2	0.14 ± 0.10
500 kGy	2.10°	48.6 ± 2.1	3.9 ± 1.1	0.42 ± 0.04
1500 kGy	3.45°	46 ± 8	−7.4 ± 4.0	0.47 ± 0.17

At 250 kGy, the crystallite size increases nominally from 63 ± 11 to 85 ± 30 nm and the microstrain changes sign; however, both changes lie within the uncertainty of the analysis, so no significant structural change of the FeGaInS_4_ crystallites can be resolved at this dose.

At 500 kGy, the crystallite size decreases to 48.6 ± 2.1 nm and the dislocation density increases to (0.42 ± 0.04) × 10^−3^ nm^−2^, accompanied by a small but significant tensile microstrain of (3.9 ± 1.1) × 10^−4^. This behavior is attributed to radiation-induced crosslinking of the polymer matrix. The radicals generated by γ-irradiation promote intermolecular bonding between neighboring PVA chains, producing a denser and more rigid network. The resulting non-uniform stresses at the filler–matrix interface, together with radiation-induced defects in the crystallites, reduce the size of the coherently diffracting domains and distort the lattice.^77^

At 1500 kGy, the crystallite size (46 ± 8 nm) and dislocation density remain at the level of the 500 kGy sample, and the microstrain does not differ significantly from zero. In contrast, the FWHM of the PVA (101) reflection, which remains between 2.1° and 2.4° up to 500 kGy, increases markedly to 3.45°, indicating a loss of crystalline order in the polymer matrix. At this dose, degradation, chain scission and amorphization of the matrix become dominant,^75,78^ whereas the FeGaInS_4_ crystallites retain the reduced domain size reached at 500 kGy.

Overall, the XRD results indicate a dose-dependent competition between crosslinking and radiation damage: crosslinking at moderate doses affects the embedded crystallites, whereas at the highest dose degradation primarily disorders the polymer matrix. Given the considerable scatter of the W–H plots, these trends should be regarded as qualitative.

### 3.3 FTIR analysis

FTIR spectroscopy was used to investigate the structural changes induced by gamma irradiation in the PVA/GO/FeGaInS_4_ nanocomposites. Fig. 5 shows the FTIR spectra of the non-irradiated sample and the samples irradiated at 250, 500, and 1500 kGy.

**Fig. 5:**
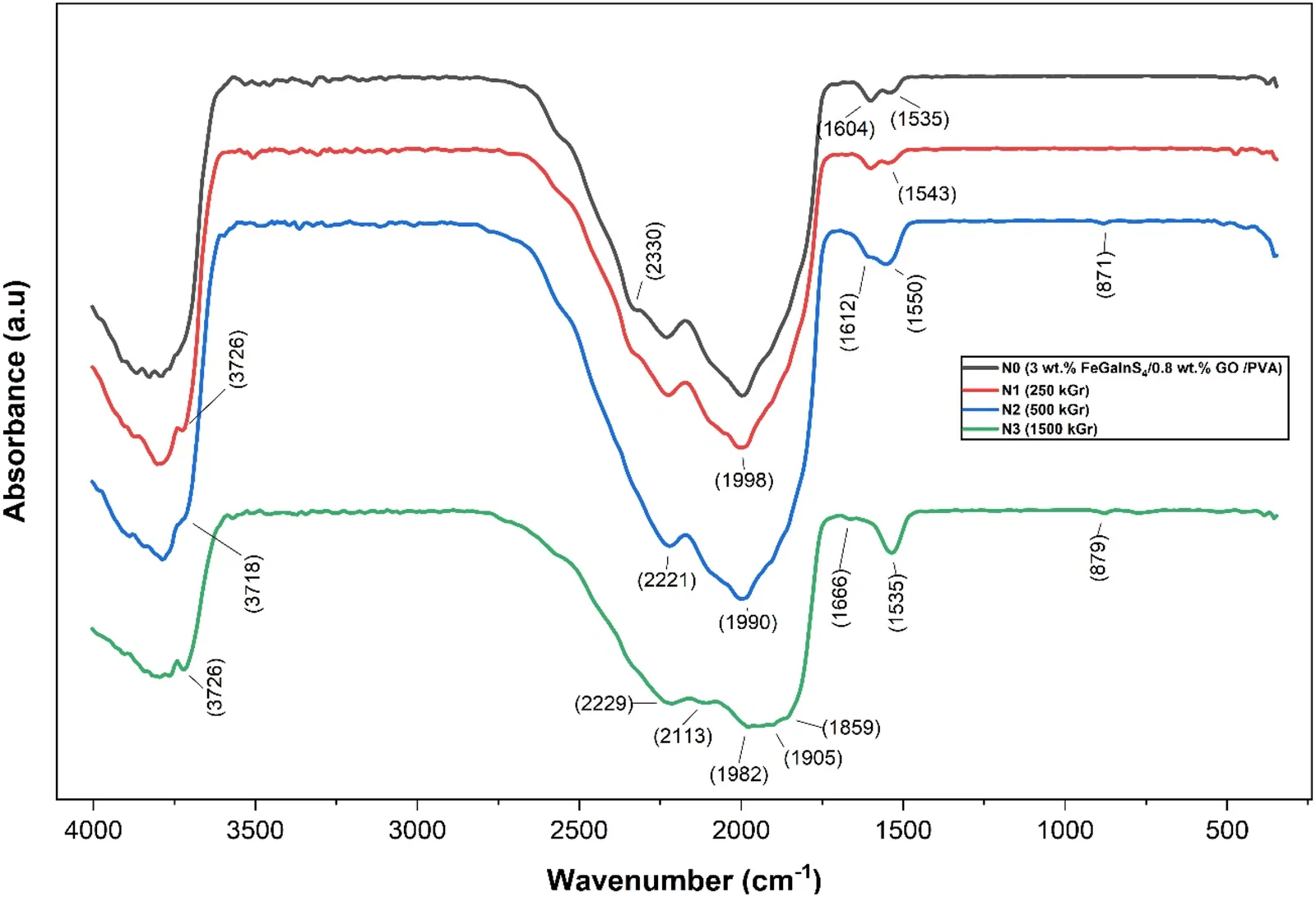
FT-IR spectra of N0, N1, N2, and N3 samples.

The broad absorption band observed in the 3726–3718 cm^−1^ region is attributed to O–H stretching vibrations originating from hydroxyl groups in the PVA matrix, oxygen-containing functional groups of GO, and adsorbed moisture. Following γ-irradiation, this band exhibits changes in position and intensity, indicating modifications in hydrogen bonding and intermolecular interactions within the composite. The shift of the O–H band toward lower wavenumbers at moderate irradiation doses suggests stronger hydrogen-bond interactions between PVA chains, GO nanosheets, and FeGaInS_4_ particles, which may be associated with the formation of a more compact and interconnected polymer network.

The absorption bands located in the 1604–1666 cm^−1^ region are assigned to overlapping contributions from adsorbed water bending vibrations, carbonyl-containing groups, and skeletal vibrations of sp^2^-hybridized carbon domains in GO. With increasing irradiation dose, these bands exhibit gradual shifts and changes in intensity, reflecting modifications in the local chemical environment of oxygen-containing functional groups. In particular, the appearance of the band near 1666 cm^−1^ in the highly irradiated sample suggests the formation of additional carbonyl species and oxidation products associated with radiation-induced degradation processes.^79^

The peaks observed near 1535, 1543, and 1550 cm^−1^ are attributed to carbon-related vibrational modes associated with graphitic domains and defect-containing structures in GO. Their dose-dependent evolution indicates irradiation-induced structural rearrangement and modification of the interfacial interactions between the polymer matrix and the filler components. At low and moderate irradiation doses, these changes are consistent with radical recombination and crosslinking processes, whereas at higher doses they indicate increasing defect concentration and structural disorder.^24,80^

Additional spectral features observed in the 1900–2330 cm^−1^ region become more pronounced with increasing irradiation dose. Although this spectral region does not correspond to the principal vibrational modes of pristine PVA, its evolution suggests the development of irradiation-induced structural modifications and changes in the chemical environment at the polymer-filler interface. The precise origin of these bands cannot be conclusively identified from FTIR analysis alone; however, their appearance is consistent with radiation-induced chemical transformations and defect formation within the nanocomposite.^25^

The weak absorption bands detected near 871–879 cm^−1^ are attributed to out-of-plane deformation vibrations of oxygen-containing carbon structures and defect-related groups associated with GO. Their slight shift after irradiation further supports modifications of the GO surface chemistry and interfacial interactions within the composite.^22,26^

Overall, the FTIR results demonstrate that γ-irradiation significantly influences the chemical structure and intermolecular bonding of PVA/GO/FeGaInS_4_ nanocomposites. At moderate irradiation doses, the observed spectral changes suggest enhanced intermolecular interactions, radical recombination, and crosslinking within the polymer network. At the highest irradiation dose, oxidation, chain scission, and defect generation become increasingly dominant.

### 3.4 Dielectric analysis

Fig. 6 shows the frequency dependence of the real part of dielectric permittivity (*ε*′) for irradiated PVA/GO/FeGaInS_4_ nanocomposites measured at different temperatures.

**Fig. 6:**
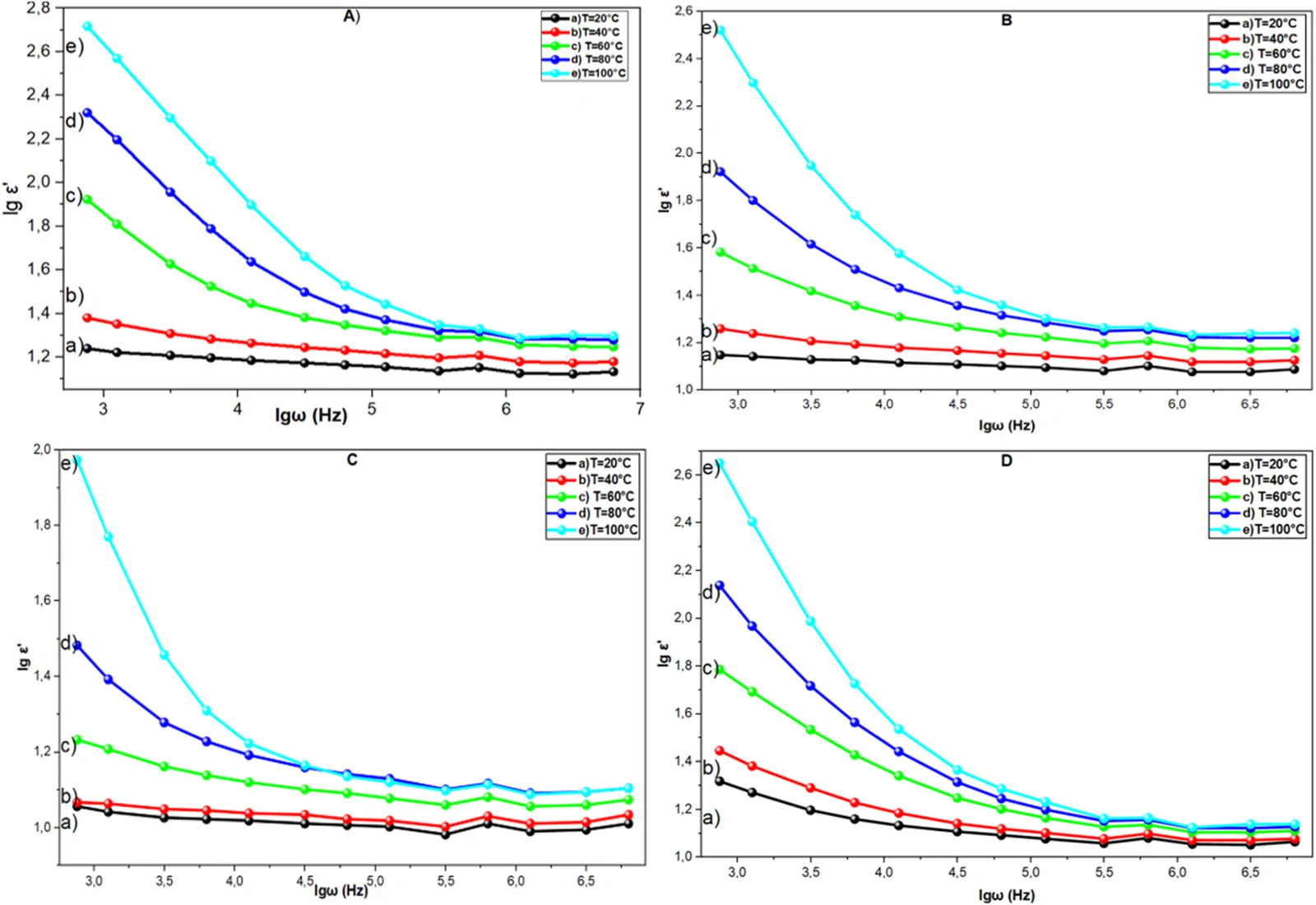
Frequency dependence of the real part of dielectric permittivity for γ-irradiated PVA-based composite films measured in the temperature range 20–100 °C: (A) 0 kGy, (B) 250 kGy, (C) 500 kGy, and (D) 1500 kGy.

For all samples, the dielectric permittivity decreases with increasing frequency. This behavior is characteristic of dipolar polarization mechanisms in polymer-based nanocomposites. At low frequencies, dipolar groups and interfacial charges can follow the alternating electric field more effectively, resulting in higher polarization and larger dielectric permittivity. However, at higher frequencies, dipoles cannot reorient rapidly enough with the applied field, leading to a gradual reduction in *ε*′.

In the PVA matrix, hydroxyl (–OH) groups are the main source of dipolar polarization.^34^ These groups also participate in intermolecular hydrogen bonding between polymer chains.^35^ As the temperature increases, partial breaking of hydrogen bonds occurs, enhancing segmental mobility of the polymer chains and facilitating dipole orientation. Consequently, the dielectric permittivity increases with temperature for all investigated samples.^36,81–83^

At relatively low temperatures (20–60 °C), the irradiated samples exposed to 250 and 500 kGy exhibit lower dielectric permittivity compared to the non-irradiated sample. This reduction can be attributed to radiation-induced crosslinking within the polymer matrix. Gamma irradiation generates free radicals, which subsequently form intermolecular crosslinks between polymer chains. The crosslinked structure restricts chain mobility^22^ and suppresses dipolar orientation,^84^ resulting in lower dielectric polarization.^75^ Notably, the decrease in *ε*′ and *σ*_ac_ at 500 kGy observed here is opposite to the behavior of the GO-free composite described in the introduction. This suggests that the GO-containing interphases promote radical-mediated crosslinking at moderate doses: the oxygen-containing groups of GO provide additional sites for radical reactions and hydrogen bonding with PVA, which restricts chain mobility and dipolar orientation.

The behavior changes significantly at the highest irradiation dose (1500 kGy). After a certain crosslinking threshold, degradation processes become dominant and polymer chain scission starts to occur. The breakdown of polymer chains increases the mobility of functional groups and dipolar entities, allowing easier dipole reorientation under the applied electric field. As a result, the dielectric permittivity increases again for the highly irradiated sample, particularly at lower temperatures.^78^

A distinct temperature-dependent behavior is observed at elevated temperatures (80–100 °C). At these temperatures, the non-irradiated sample exhibits the highest dielectric permittivity, whereas the irradiated samples show comparatively lower values. This phenomenon may be associated with radiation-induced oxidative processes. Gamma irradiation promotes the formation of free radicals, which can react with oxygen to produce hydroperoxide and carbonyl-containing species.^26,79^ At elevated temperatures, these oxygen-containing groups undergo further structural transformation and decomposition, generating heavier oxygen-based functional groups that hinder dipolar motion. Consequently, the mobility of dipolar units decreases at high temperatures, leading to reduced dielectric permittivity in highly irradiated samples.^85^

Furthermore, the strong decrease of *ε*′ in the low-frequency region suggests the contribution of interfacial or Maxwell–Wagner–Sillars polarization arising from charge accumulation at the interfaces between PVA, GO, and FeGaInS_4_ fillers. As the frequency increases, this interfacial polarization gradually diminishes, and the dielectric response becomes dominated by faster electronic and atomic polarization mechanisms.

Fig. 7 presents the frequency dependence of the displacement current density (*J*_d_) for the investigated composites.

**Fig. 7:**
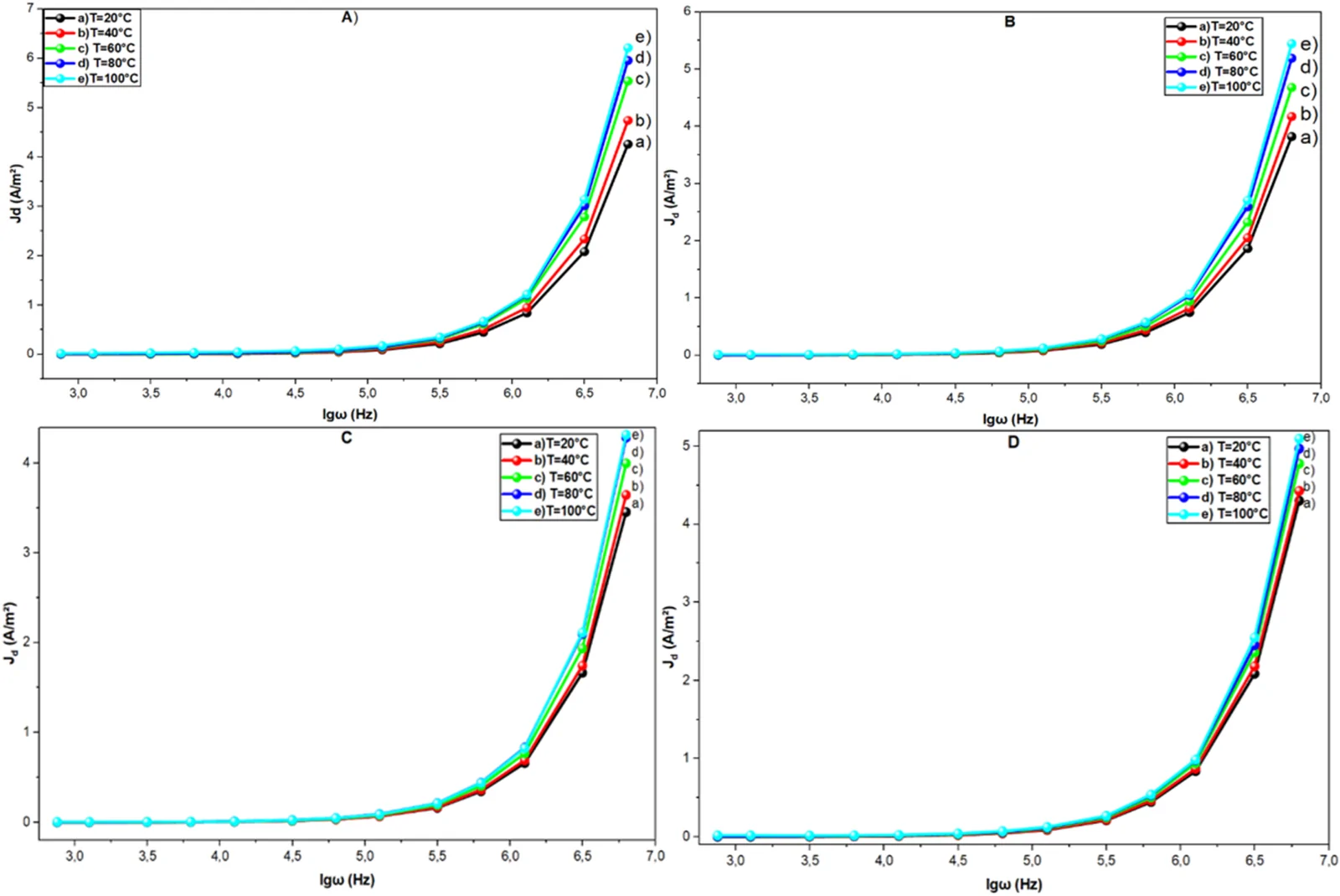
Frequency dependence of the displacement current density (*J*_d_) for γ-irradiated PVA-based composite films measured in the temperature range 20–100 °C: (A) 0 kGy, (B) 250 kGy, (C) 500 kGy, and (D) 1500 kGy.

Fig. 7(A–D) presents the frequency dependence of the displacement current density (*J*_d_) of γ-irradiated PVA/GO/FeGaInS_4_ composites at different temperatures for irradiation doses of 0, 250, 500, and 1500 kGy, respectively. For all samples, (*J*_d_) increases with increasing frequency. This behavior is associated with the direct proportionality of the displacement current to frequency and the increasing rate of variation of the applied electric field. At low frequencies ((10^3^–10^5^) Hz), (*J*_d_) exhibits relatively small values and the effect of temperature is weak. As the frequency increases, the contributions of dipolar polarization, interfacial charge accumulation, and hopping transitions of localized charge carriers become more pronounced.

An increase in temperature leads to a rise in the displacement current density for all samples. This behavior can be attributed to the thermal activation of dipoles, enhanced segmental mobility of polymer chains, and an increased probability of charge carrier transitions between localized states. The greater separation of the curves at higher frequencies indicates that the polarization processes are strongly temperature dependent.^81,83^

A reduction in (*J*_d_) is observed for the samples irradiated at 250 and 500 kGy compared with the unirradiated composite. This behavior may be associated with the predominance of crosslinking processes at intermediate irradiation doses. The formation of crosslinks restricts the mobility of polymer chains, hinders dipole orientation, and reduces the intensity of interfacial polarization processes. As a consequence, the displacement current density decreases.

In contrast, the sample irradiated at 1500 kGy exhibits a renewed increase in (*J*_d_). This behavior can be attributed to structural degradation occurring at high irradiation doses, including chain scission, the formation of localized defect centers, and enhanced interfacial charge accumulation. Such structural modifications strengthen the response of dipoles and charge carriers to the applied electric field, resulting in enhanced polarization capability and higher displacement current density values.^78,86^

Therefore, the influence of γ-irradiation on the displacement current density exhibits a two-stage behavior. At low and intermediate doses, crosslinking processes dominate and lead to a decrease in (*J*_d_), whereas at high doses, polarization mechanisms associated with defect formation and structural degradation become dominant, resulting in an increase in (*J*_d_).

Since *J*_d_ is directly related to the dielectric polarization of the material, its variation reflects radiation-induced changes in polarization and dielectric relaxation processes. At all irradiation doses, *J*_d_ increases with increasing frequency and temperature. The observed changes indicate that gamma irradiation significantly modifies the polarization behavior of the polymer composite through structural rearrangements occurring within the polymer matrix and at the filler–matrix interfaces.

The frequency dependence of dielectric loss (tan *δ*) for irradiated PVA/GO/FeGaInS_4_ nanocomposites at different temperatures is shown in Fig. 8.

**Fig. 8:**
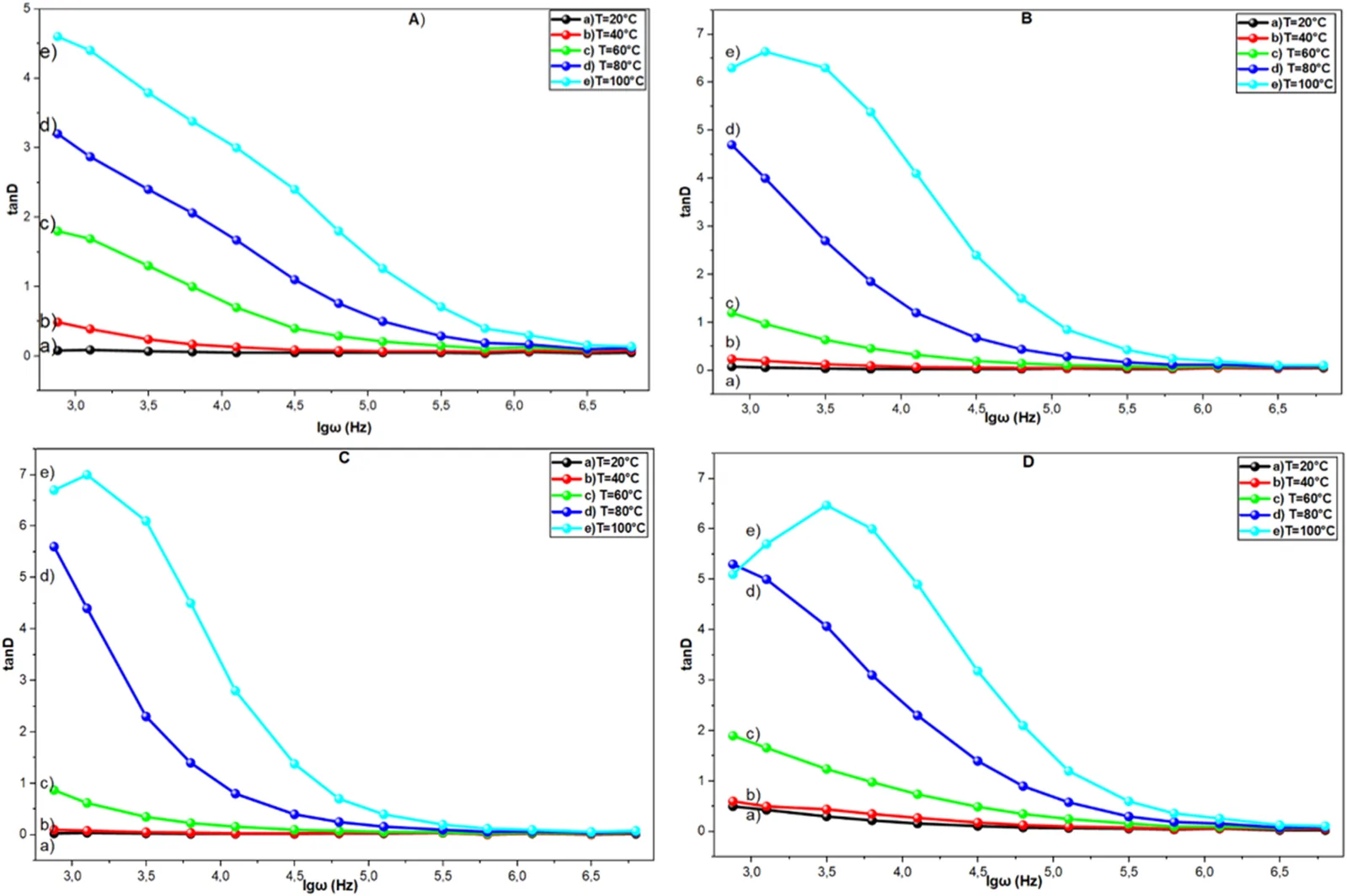
Frequency dependence of the dielectric loss for γ-irradiated PVA-based composite films measured in the temperature range 20–100 °C: (A) 0 kGy, (B) 250 kGy, (C) 500 kGy, and (D) 1500 kGy.

Similar to the dielectric permittivity behavior, dielectric loss increases with temperature and decreases with increasing frequency for all investigated samples. This behavior is associated with dipolar polarization and relaxation processes within the polymer matrix.^81,84^

At low frequencies, dipoles and interfacial charges are able to follow the alternating electric field more effectively, resulting in higher energy dissipation and larger dielectric loss values. As the frequency increases, dipoles cannot orient rapidly enough with the applied field, leading to a decrease in tan *δ*.^40,83^

At relatively low temperatures (20, 40, and 60 °C), the samples irradiated with 250 and 500 kGy exhibit lower dielectric loss compared to the non-irradiated sample. This behavior can be attributed to radiation-induced crosslinking within the polymer matrix. Gamma irradiation generates free radicals that promote intermolecular crosslink formation, restricting polymer chain mobility and suppressing dipolar motion. As a result, dielectric relaxation and energy dissipation decrease.^41^

With increasing temperature, hydrogen bonds within the PVA matrix gradually weaken and partial bond breaking occurs, allowing dipoles and polymer segments to move more freely. Consequently, dielectric loss increases due to enhanced dipolar relaxation and increased molecular mobility.

The sample irradiated at the highest dose (1500 kGy) demonstrates a different behavior. At lower temperatures, tan *δ* increases compared to the moderately irradiated samples. This increase is associated with irradiation-induced chain scission and degradation processes, which enhance the mobility of dipolar groups and facilitate dielectric relaxation. The formation of free radicals and structural disorder contributes to increased energy dissipation under the alternating electric field.^86^

However, at elevated temperatures (80 and 100 °C), the dielectric loss of the highly irradiated sample decreases. This behavior may be related to radiation-induced oxidation processes and the formation of oxygen-containing functional groups such as carbonyl and peroxide species. These groups can act as localized trapping centers and restrict dipolar orientation under the applied electric field, thereby suppressing dielectric relaxation and reducing tan *δ* at high temperatures.^18,85^

A noticeable shift of the tan *δ* maximum toward higher frequencies is observed with increasing irradiation dose and temperature. The dielectric loss peak appears when the frequency of the applied electric field approaches the characteristic relaxation frequency of the system. Since the relaxation time (*τ*) is inversely proportional to relaxation frequency, the shift of the peak toward higher frequencies indicates a reduction in relaxation time.^81,84,87^

In the non-irradiated sample at low temperatures, the relaxation peak is not clearly observed within the measured frequency range because the relaxation process occurs at lower frequencies corresponding to longer relaxation times. After irradiation and temperature increase, the relaxation time decreases due to enhanced molecular mobility, defect formation, and structural modifications, causing the relaxation peak to shift into the measurable frequency region.

The reduction of relaxation time may initially be associated with chain scission and free-radical formation induced by irradiation. Although crosslinking becomes significant at intermediate irradiation doses, increasing temperature partially weakens intermolecular hydrogen bonding and increases segmental freedom within the polymer matrix. At higher irradiation doses, degradation effects become more dominant, resulting in additional bond breaking and enhanced molecular mobility. Consequently, the relaxation dynamics accelerate and the relaxation time decreases further.

Fig. 9 shows the frequency dependence of the electrical conductivity of the gamma-irradiated PVA-based composite films at different temperatures.

**Fig. 9:**
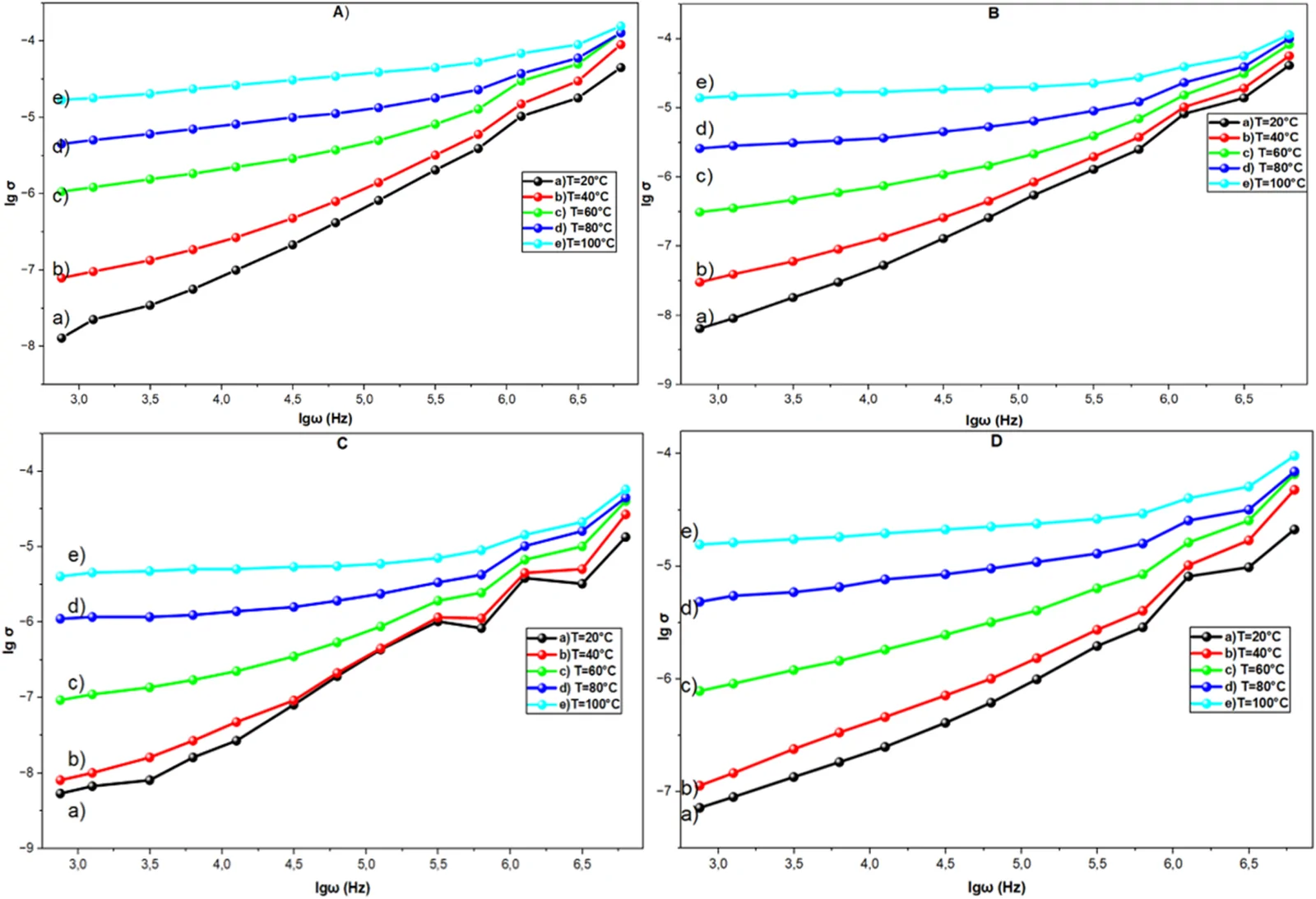
Frequency dependence of the electrical conductivity for γ-irradiated PVA-based composite films measured in the temperature range 20–100 °C: (A) 0 kGy, (B) 250 kGy, (C) 500 kGy, and (D) 1500 kGy.

The electrical conductivity increases with both increasing temperature and frequency for all investigated samples. The temperature dependence indicates a thermally activated charge transport process, while the increase in conductivity with frequency is characteristic of hopping conduction in disordered polymer systems. The conductivity decreases at irradiation doses of 250 and 500 kGy, suggesting that radiation-induced crosslinking is the dominant process in this dose range. The formation of intermolecular crosslinks restricts charge carrier mobility and increases the energy barrier for hopping transport. In contrast, the maximum conductivity is observed at 1500 kGy. At this irradiation dose, chain scission processes become dominant, generating additional localized states and charge transport pathways. Furthermore, the possible partial reduction of graphene oxide and the formation of radiation-induced defects may contribute to the enhancement of electrical conductivity. The values of *W*_M_ listed in Table 6 were obtained from eqn (11) using the frequency exponent *s* determined at *T* = 293 K and *f* = 5 kHz, with *τ*_0_ = 10^−13^ s (inverse of a typical phonon frequency).^54^*R*_ω_ and *N* were subsequently obtained from eqn (9) and (10), respectively, using the value of *ε*′ measured at the same temperature and frequency.

**Table 6:** Parameters of the correlated barrier hopping model for the PVA/GO/FeGaInS_4_ composites, determined at *T* = 293 K and *f* = 5 kHz (*τ*_0_ = 10^−13^ s)

Sample	*s*	*R*_ω_, nm	*N*, m^−3^ × 10^27^	*W*_M_, eV
N0	0.750 ± **0.04**	0.50 ± **0.01**	2.12 ± **0.05**	1.10 ± **0.04**
N1	0.805 ± **0.07**	0.48 ± **0.015**	1.95 ± **0.07**	1.27 ± **0.05**
N2	0.845 ± **0.05**	0.52 ± **0.012**	1.06 ± **0.021**	1.47 ± **0.06**
N3	0.660 ± **0.065**	0.87 ± **0.02**	0.57 ± **0.085**	0.94 ± **0.07**

It can be seen from the table that the *s* parameter increases at low and moderate irradiation doses and then decreases at 1500 kGy, indicating a dose-dependent change in the charge transport mechanism. The increase in *s* from N0 to N2 suggests that the correlated barrier hopping (CBH) mechanism is dominant in the low- and moderate-dose range. In this region, gamma irradiation promotes radical-induced crosslinking between polymer chains, producing a denser and more rigid polymer network. This crosslinked structure restricts segmental mobility, reduces the freedom of charge carriers, and increases the potential barrier height for hopping transport. As a result, the electrical conductivity decreases at 250 and 500 kGy.

At the highest irradiation dose of 1500 kGy, the *s* parameter decreases markedly, while the potential barrier height also becomes lower. This behavior indicates that chain scission, degradation, amorphization, and defect formation become dominant at high irradiation dose. These structural changes generate additional localized states and charge transport pathways, facilitating carrier movement through the composite. The decrease in *s* suggests that the conduction mechanism tends to shift from CBH-dominated hopping toward quantum mechanical tunneling (QMT)-assisted transport.^52,54^

The decrease in *N* with irradiation dose may be associated with the transformation of localized states into deeper trapping centers or with the partial elimination/rearrangement of shallow traps during irradiation. Therefore, although the effective density of localized states decreases, the reduced barrier height and radiation-induced structural disorder at 1500 kGy enhance charge transport. Thus, the conductivity behavior confirms that low and moderate irradiation doses are dominated by crosslinking-induced transport suppression,^85,86^ whereas high-dose irradiation promotes defect-assisted and QMT-type conduction.^86^

Table 7 presents the activation energies calculated at different frequencies for PVA-based composites exposed to various gamma irradiation doses.

**Table 7:** Activation energy of PVA-based composites exposed to different gamma irradiation doses

Sample	500 Hz	2 kHz	20 kHz	50 kHz
N0	0.78 ± 0.09	0.69 ± 0.016	0.47 ± 0.035	0.37 ± 0.069
N1	0.76 ± 0.068	0.65 ± 0.075	0.48 ± 0.047	0.4 ± 0.078
N2	0.74 ± 0.054	0.60 ± 0.056	0.3 ± 0.023	0.21 ± 0.085
N3	0.56 ± 0.07	0.50 ± 0.042	0.36 ± 0.068	0.29 ± 0.045

The calculated activation energy values reveal that, at low frequencies, the activation energy decreases with increasing irradiation dose. As discussed previously, the charge transport mechanism in the low-frequency region is mainly governed by the correlated barrier hopping (CBH) model. Gamma irradiation induces structural modifications in the polymer matrix, leading to the formation of new localized states and reducing the distance between existing localized states. As a result, electrons require lower thermal energy to hop between neighboring trapping sites.

At higher irradiation doses, particularly at 1500 kGy, the decrease in activation energy becomes more pronounced. This behavior may be associated with irradiation-induced annealing of certain trapping centers and a reduction in trap depth. The formation of additional defects and structural disorder also contributes to enhanced charge carrier mobility and easier hopping transitions.^85^

As the frequency increases, the activation energy decreases further. This phenomenon is related to the inability of dipoles and charge carriers to follow the rapidly alternating external electric field at high frequencies. In other words, at high frequencies the electric field changes too rapidly for charge carriers to thermally overcome the potential barriers within a single field cycle, and the hopping process cannot be completed effectively.

Under these conditions, however, the probability of electron tunneling increases. A high-frequency AC electric field periodically modifies the potential barrier profile. When the field becomes positive, one side of the barrier is lowered, whereas the opposite side decreases during the negative half-cycle. Consequently, the barrier acquires an asymmetric shape and its effective width periodically decreases. Due to this asymmetry and barrier narrowing, the decay of the electron wave function inside the barrier becomes weaker, which exponentially enhances the tunneling probability.

Therefore, at high frequencies, quantum mechanical tunneling (QMT) gradually becomes the dominant charge transport mechanism instead of thermally activated hopping conduction. This transition is accompanied by a reduction in activation energy.

As seen in the table, an anomalous behavior is observed at higher frequencies depending on irradiation dose. At 250 kGy, the activation energy initially increases compared to the non-irradiated sample. This behavior may be related to the onset of crosslinking processes. Free radicals generated by irradiation begin to form new intermolecular bonds between neighboring polymer chains, producing a denser and more compact structure. Such structural densification enhances the interaction between trapped electrons and surrounding ionic groups of the polymer matrix. Consequently, the effective potential barrier experienced by charge carriers increases, requiring higher energy for electron transport.

With increasing irradiation dose, the degree of crosslinking becomes stronger and localized trapping centers approach each other more closely, facilitating tunneling transitions and reducing activation energy. However, at very high doses, degradation and chain scission become dominant. The trapping centers become more separated due to structural disorder, while some shallow traps are annealed or eliminated by irradiation. As a result, activation energy may increase again in certain frequency regions.^18,78^

The conductivity behavior is consistent with these structural modifications. The conductivity decreases at 250 and 500 kGy, which may be attributed to crosslinking-induced restriction of charge carrier mobility. In contrast, the conductivity increases at 1500 kGy due to degradation, chain scission, and partial annealing of trapping centers, which enhance carrier transport within the polymer nanocomposite system.

## 4 Conclusion

The effects of γ-irradiation on the structural, spectroscopic, dielectric and electrical properties of dual-filler PVA/GO/FeGaInS_4_ nanocomposites were investigated in the dose range of 0–1500 kGy. The results show that irradiation modifies the composite structure and charge transport through a dose-dependent competition between crosslinking and degradation processes.

XRD analysis confirmed the structural stability of the FeGaInS_4_ crystalline phase after irradiation, while the absence of the characteristic GO diffraction peak indicated the exfoliated dispersion of graphene oxide within the polymer matrix. Williamson–Hall analysis showed that the FeGaInS_4_ crystallite size remained unchanged within experimental uncertainty at 250 kGy and decreased to about 47 nm at 500 and 1500 kGy, accompanied by an increase in dislocation density. The broadening of the PVA (101) reflection at 1500 kGy indicated a loss of crystalline order in the polymer matrix.

FTIR spectroscopy demonstrated irradiation-induced modifications in hydrogen bonding and oxygen-containing functional groups. The shift of the O–H stretching band suggested enhanced intermolecular interactions at moderate irradiation doses, whereas the appearance of additional carbonyl-related features at high doses indicated oxidation and degradation processes.

EPR spectroscopy confirmed the formation of radiation-induced radicals, with the highest radical concentration at 250 kGy and lower concentrations at higher doses, indicating that recombination and participation of radicals in crosslinking reactions become increasingly important as the dose increases. During 90 days of storage, the radical signal decreased in all irradiated samples. The decay was fastest at 250 kGy, consistent with bimolecular recombination, and slowest at 1500 kGy, which points to comparatively stable, trapped paramagnetic centers, possibly associated with defects in partially reduced GO or with conjugated radicals in the polymer matrix. In contrast, the broad Fe-related resonance remained unchanged, confirming the structural stability of the FeGaInS_4_ phase under irradiation.

Dielectric measurements showed that the permittivity, dielectric loss and displacement current density decrease at 250 and 500 kGy, reflecting restricted dipolar motion caused by irradiation-induced crosslinking, whereas the sample irradiated at 1500 kGy exhibits an enhanced dielectric response associated with chain scission and defect formation. The ac conductivity follows the same trend, with the lowest values at moderate doses and the highest at 1500 kGy. The decrease in permittivity and conductivity at moderate doses is opposite to the behavior of the GO-free FeGaInS_4_/PVA system, indicating that the GO-containing interphases promote crosslinking in the dual-filler composite.

Analysis of the ac conductivity revealed that the frequency exponent *s* increased from 0.75 for the non-irradiated sample to 0.84 at 500 kGy and then decreased to 0.66 at 1500 kGy, while the hopping barrier height increased from 1.10 to 1.47 eV and then decreased to 0.94 eV. These results indicate that correlated barrier hopping dominates charge transport in the crosslinked structure, whereas high-dose irradiation promotes defect-assisted transport with an increasing contribution of quantum mechanical tunneling. At low frequencies, the activation energy decreases with increasing dose, reflecting the formation of radiation-induced localized states.

Overall, γ-irradiation induces a transition from a crosslinking-dominated regime at moderate doses (250–500 kGy) to a degradation-dominated regime at 1500 kGy. Moderate doses produce a denser and more constrained polymer network that suppresses dielectric losses and charge transport, whereas high doses generate structural defects, additional localized states and enhanced polarization pathways. γ-Irradiation can therefore be used to tailor the dielectric and electrical properties of PVA/GO/FeGaInS_4_ nanocomposites. Orientation-resolved measurements and additional doses between 500 and 1500 kGy are expected to clarify the respective roles of the matrix and the oriented filler interfaces and to locate the transition between the two regimes more precisely.

## Author contributions

Zeynab Addayeva – investigation, data curation, writing – original draft, writing – review & editing, project administration. Goncha Eyvazova – software, visualization. Namiq Niftiyev – software, data curation. Faiq Mammedov – resources. Rasim Aslanov – resources, software, visualization. Oktay K. Gasymov – resources. Nahida Musayeva – resources, software, visualization. Hokman Mahmudov – resources. Mustafa Muradov – conceptualization, methodology, validation, supervision.

## Conflicts of interest

The authors declare no competing interests.

## Data Availability

The datasets generated and/or analysed during the current study are available from the corresponding author on reasonable request.
